# Analysing the factors that influence social media adoption among SMEs in developing countries

**DOI:** 10.1007/s10843-023-00330-9

**Published:** 2023-04-25

**Authors:** Offiong Helen Solomon, Tom Allen, Wangari Wangombe

**Affiliations:** 1 University of Westminster, London, NW1 5LS UK; 2 De Montfort University, The Gateway, Leicester, LE19BH UK

**Keywords:** Social media adoption, SMEs, Africa, L20, M3, O14, O33

## Abstract

Social media penetration is on the rise in developing countries and is an important channel of growth for small and medium enterprises (SMEs). Many SMEs in developing countries use social media to connect their customers to their products and services. However, the factors that have led the existing SMEs in Africa to adopt or reject social media need to be clarified to understand the key contributing factors and influences at play. This paper adopts the learning-by-doing concept from economic theory to explore the factors that influence the adoption of social media. A primary survey follows this to examine the use of social media among firms in the commercial districts of Kenya and Nigeria. The preliminary surveys in both countries were combined into a single dataset to analyse the relationship between social media use and learning-by-doing.

The results show that while small SMEs are limited in their social media capacity, medium size firms tend to invest in their social media presence, and larger-size firms still rely on traditional marketing channels.

**Supplementary Information:**

The online version contains supplementary material available at 10.1007/s10843-023-00330-9.

## Abstract (es)

La penetración de las redes sociales va en aumento en los países en desarrollo y podría ser un canal de crecimiento importante para las pequeñas y medianas empresas (PYMES). En consecuencia, muchas pymes de países en desarrollo utilizan las redes sociales para conectar a sus clientes con sus productos y servicios. Sin embargo, ha habido una investigación limitada sobre los factores que influyen en la adopción de las redes sociales en los países en desarrollo de África. Nuestro artículo adopta el concepto de aprender haciendo de la teoría económica para explorar los factores que influyen en la adopción de las redes sociales. A esto le sigue una encuesta primaria para explorar el uso de las redes sociales entre las empresas en los distritos comerciales de Kenia y Nigeria. Las encuestas primarias en ambos países se combinaron en un solo conjunto de datos para analizar la relación entre el uso de las redes sociales y el concepto de aprender haciendo. Nuestros resultados muestran que el tiempo y el conocimiento invertido en aprender a desarrollar una presencia en las redes sociales aumentan la probabilidad de adopción de las redes sociales. Es más probable que las empresas más pequeñas adopten las redes sociales para internacionalizarse, mientras que las empresas más grandes que pueden invertir en las redes sociales tienden a utilizar canales de marketing más tradicionales.

## Summary highlights

*Contributions.* This paper focuses on SME’s ability to adopt social media and the role of learning-by-doing in helping SME’s innovate. This contribution is especially necessary having observed that literature on social media adoption among SMEs has focused on end-users rather than the efficacy of adoption by SMEs, creating a deficit in the literature on social media adoption among SMEs (Durkin et al., 2013). This fact is heightened and made more apparent in the wake of the COVID-19 pandemic and the government’s response to this (Almotairy et al., 2020).

*Purpose/research questions.* This study has five main objectives: (1) to add to the current literature from the perspective of SMEs; (2) to unravel the decision making process, reasoning, and criteria of adoption and use of social media platforms by SMEs; (3) to highlight the extent of use of the platforms adopted for various business operations; (4) to consider the determinants of the efficacy of use, such as training, interest, experience, and exposure and finally; (5) to determine how effective use of social media was in generating sales, increasing exposure and market share, as well as determining what the market conditions are. To achieve this, a primary survey of local SMEs in Kenya and Nigeria was conducted to gauge business attitudes on the use of social media in their business. This was undertaken by administering a questionnaire to 100 local SMEs in Lagos and Nairobi. The study applies a binary logistic regression model to estimate the likelihood of firms using social media to promote sales and applies control variables that capture firm characteristics.

*Findings/results.* The results are then organised in sections covering various questions asked, for example, questions such as why enterprises in Lagos and Nairobi used social media were asked and results helped uncover how SMEs in Kenya and Nigeria use social media, and its impact on sales. The study similarly finds that several variables affect social media adoption, including knowledge of social media platforms as well as the customer’s presence on social media (Bogea and Brito, 2018). In particular, observations are made showing that smaller firms use social media more than larger firms for the primary function of facilitating advertising and marketing campaigns as well as gathering information from customers. Data showed that in Kenya, 43% of firms used Facebook, 32% WhatsApp, and considered spending time on social media a crucial aspect to increasing sales. Statistics shown in Table 1 indicate that time invested in learning how to use social media contributes significantly to the accumulation of knowledge in social media, and learning-by-doing are of business strategic importance and significant to business success. This finding is complemented by a robustness test as shown in Table 3 which shows that service-based urban SMEs in Kenya and Nigeria willing to adopt social media have their knowledge of using social media tools increasing with time invested in using the technology. Consequently, this paper finds that training is recommended to aid business owners in using social media effectively.

**Table 1:** Summary statistics for the analysis sample

Variable	Description	Obs	Mean	Standard deviation	Min	Max
*S*	Social media adoption	182	0.74	0.44	0	1
*K*	Knowledge of social media	197	2.65	0.60	1	3
*B*	Business Strategy importance	196	3.76	0.53	1	3
*H*	Time invested in using social media	186	4.11	0.80	1	5
Age	Age of business	187	0.36	0.48	0	1
Size	Firm size	184	0.15	0.36	0	1
Country	Countries in the sample	199	0.50	0.50	0	1

N.B. We tested the sample and found no multicollinearity. Results available from the author

*Limitations.* We recognise that due to the sample size, and selection, sample is not completely representative and that these findings may vary across region, both geographically and demographically. This is primarily owing to limited resources, confining the study samples to the two cities Lagos (Nigeria) and Nairobi (Kenya), areas and regions outside of these, thus the results cannot be considered generalised to rural areas where there are more technological barriers. Second, the survey covers a short period using 100 usable responses in Nairobi and 99 usable responses in Lagos. This means the results reveal a snapshot of the factors that influence social media adoption at a point of time. While this study still accomplishes the goals as identified by the study objectives such as that of adding to the literature consequently, a larger study over a longer duration would further facilitate the analysis of social media adoption among SME’s in developing countries, enabling a more robust comparison.

*Theoretical implications and recommendations.* This study highlights the role of learning and knowledge of social media as a cause of internationalisation among firms, particularly SME’s in order to compete effectively at a macro scale. The authors recommend that further research on the role of knowledge in amplifying the use of social media as a business tool will help determine the channels by which social media contributes to increasing sales.

*Practical implications and recommendations.* This study accomplishes the goal of adding to the literature and thereby provides a starting point for practitioners to understand how SME entrepreneurs in developing countries use social media to internationalise their business operations which can help in exposing potential opportunities

## Introduction

This paper addresses the role of knowledge and learning-by-doing as factors that influence social media adoption among SMEs in developing countries. According to Durkin et al. (2013) and, more recently, Almotairy et al. (2020), there has been a deficit in the literature on social media adoption among SMEs. This is especially the case in developing countries because collecting primary data on how SMEs use social media for business promotion is challenging. The need for research in this area has increased in urgency due to the effects of the COVID-19 pandemic and the government’s response, which have seriously impacted the business landscape and placed a much greater emphasis on digital business. Second, social media has increasingly become necessary (Cesaroni and Consoli, 2015), especially among young SMEs and aspiring entrepreneurs in emerging markets. The low-cost adoption of these technologies helps these SMEs to increase their visibility, reach consumers and other businesses, and internationalise, thereby increasing access to potential cross-border markets (Haller and Siedschlag, 2011). However, the internalisation of SMEs depends on their ability to adopt social media effectively and take advantage of this technology in the global economy.

Despite the growing interest in social media technology, the focus of the literature has been on end-users (Choi and Thoeni, 2016) rather than an SME context in terms of the perceived factors that influence social media adoption. The limited literature on the diffusion of social media adoption in developing countries is further supported by Wamba and Carter (2016). The ability of SMEs to adopt social media into their daily business depends on factors such as technological knowledge (Dahnil et al., 2014). To the best of our knowledge, there has yet to be an attempt to develop a theoretical framework for identifying factors influencing the likelihood of social media adoption, particularly the role of learning-by-doing.

The focus on SMEs is driven by observations from our primary data showing that they recognise the global business opportunities of using social media to complement their ‘personal contact networking activity’ with customers (Kaplan and Haenlein, 2010). Furthermore, the observations from our primary data on Kenya and Nigeria show that some SMEs use social media relatively more frequently than others. Consequently, it is essential to understand the factors that explain differences in the adoption of SMEs. This will help businesses strategically use this technology to quickly reach their potential customers (Ahmad et al., 2018; Baby and Joseph, 2016) and improve business performance.

Evidence suggests that social media penetration is rising faster in developing countries than in developed countries (Dixon, 2023), creating new opportunities for local businesses to advertise for selling purposes. However, there needs to be more literature on the factors driving local businesses in Africa to adopt social media.

The theoretical focus on learning-by-doing is motivated by Parrilli and Elola (2012), who argue that learning-by-doing is critical in helping small businesses innovate. As an organisational strategy, small businesses that adopt information technology (IT) are more likely to have CEOs who are knowledgeable and positively disposed to adopt technology (Thong and Yap, 1995). Regarding firms’ characteristics, SMEs are more likely to use social media sites because they have limited time and financial resources. Jones et al. (2015) found that social media increased the ability of small businesses in Maine, USA, to build relationships with existing customers and reach new customers. The lack of working capital in Africa constrains how much small businesses can market and advertise their products using traditional platforms, such as TV and print media. Therefore, it has been argued that social media can provide low-cost alternatives for small businesses to create and promote engagement with their products to expand customer outreach (Batta, 2018).

Using low-cost technology, such as social media, is particularly attractive to young entrepreneurs driven to internationalisation. It is essential for new entrants who must carve a niche in a highly competitive domestic market. Arafat et al. (2022) found that technology use was directly proportional to internalisation by local businesses. They also found that intense domestic competition leads to increased internationalisation among entrepreneurs, particularly new ventures.

This paper surveys the status of social media adoption among local businesses in Kenya and Nigeria. According to the Chakravoti et al. (2015) in the Harvard Business Review, Kenya and Nigeria were classified as ‘breakout’ countries because they have the potential to become strong digital economies in the future. Hence, this research focuses on the locus of these two countries. We find evidence from the analysis of this survey data that knowledge is an essential component of social media adoption and that learning has a role in enhancing that knowledge base. Furthermore, businesses that identify social media adoption as a crucial part of their business strategy are likelier to use this channel.

The rest of the paper follows: the ‘Conceptual framework’ section presents a theoretical framework grounding our hypotheses. The ‘Survey design’ section presents the main findings of our survey. The ‘Data’ section describes the data and empirical framework. The ‘Results and discussion’ section presents and discusses the results. The ‘Conclusion’ section offers the conclusion.

## Conceptual framework

For many businesses, social media presence is now a standard feature of their operations. Several authors have investigated why this would be so. What precisely fits a firm from developing a social media presence? One answer is that social media is beneficial to overall business performance. In a recent review of the literature, Almotairy et al. (2020) found that existing research points to a positive relationship between social media use and the performance of start-up businesses. This success is moderated by variables such as the gender and age of the business owner (Karikari and Owusu-Frimpong, 2017). The sources of this general positive effect have been identified in further detail. Hur et al. (2017) found that for small businesses, social media provides an environment that helps them develop market knowledge and share information with customers.

Other authors found that the use of social media contributes to knowledge sharing (Munar and Jacobson, 2014), promoting brand awareness (Nisar and Whitehead, 2016) and developing customer relationships (Rosman and Stuhura, 2013; Qalati et al., 2021). Kumar et al. (2013) found that social media contributes to sales growth via word-of-mouth information sharing of the brand experience. This is argued to be of particular importance for small firms with limited marketing budgets. Borah et al. (2022) find that social media usage by SMEs has a positive effect on innovation capabilities and performance. Rodrigues Gutierrez et al. (2022) also found evidence from surveying business-to-business salespeople in various industries that social media positively influences sales processes. There can also be downsides to social media use, one of which was identified by Golmohammadi et al. (2021), who showed that by using social media to deal with customer complaints, firms might inadvertently highlight the complaint, and this may generate an adverse effect that is larger than any positive effect from being willing to deal with complaints.

In summary, there is substantial evidence that the use of social media can enhance the performance of businesses across a variety of functions, and this has been given greater prominence during the COVID-19 pandemic and related government restrictions.

If it is true that using social media can benefit businesses, then it follows that using social media well would be even more beneficial. Companies capable of using social media well would be more likely to adopt it as part of their business strategy. This begs the question: Which businesses will be more likely to use social media productively? We argue that businesses with more knowledge will be better at using it. The cost to a business of successfully using social media includes both direct financial costs and the opportunity cost of the time involved by the owner, which may be especially valuable for small businesses with otherwise scarce resources. Both implicit and explicit costs can be reduced by a more knowledgeable user who can direct resources into more productive avenues. Various literature supports this intuitive idea that knowledge about a business process is essential. Trieu et al. (2022) put this idea into the context of information systems, applying the ‘theory of effective use’ to show that knowing what it takes to use business information systems is very important.

Specific evidence of this effect in business innovation and social media use includes Vuorio et al. (2020), who found that human capital is a broadly determining factor in service innovation. Belitski et al. (2019) argued in their survey that ‘successful entrepreneurship is affected by the speed and efficiency of new knowledge integration into firms and industry routines’. Gutierrez et al. (2022) looked specifically at social media use, finding that a positive effect on firm performance is more likely with a younger manager, which they linked to knowledge of social media. Tajvidi and Karami (2017) surveyed the UK hotel industry. They found a positive relationship between social media use and firm performance. However, marketing capability was found to be a significant mediating factor. Again, we see the argument that social media use alone can only go so far. Knowing what you are doing will make your production of a social media presence more effective. Finally, Oggero et al. (2020) emphasised the role of digital skills in promoting entrepreneurship. There is a considerable body of theoretical and empirical literature to support the intuition that knowledge about a process such as using social media will increase the effectiveness of businesses. Therefore, we propose the first hypothesis:

Hypothesis 1 (H1): A high level of knowledge about social media is positively related to an SME’s adoption of social media for business operations.

If this hypothesis is correct, a follow-up question would be, where does a high level of knowledge come from? One idea comes from the economics literature, specifically the concept of ‘learning-by-doing’. The simple intuition captured in models such as those of Dasgupta and Stiglitz (1988) is that the more a firm does something, the more they learn about this process and the more efficient they become at doing it. This point is typically made in the context of the primary productive function of the firm but makes just as much sense in terms of other processes, such as advertising and the development of a social media presence; the more experience you have, the more you learn, and the better you get at it. Evidence for this kind of effect can be found in Irwin and Klenow (1994), who found evidence of learning-by-doing effects in the semiconductor industry. Irwin (2000) also examines learning effects in the nineteenth-century US steel industry. He found that these learning effects are modestly significant and flow fairly easily internationally. Huang et al. (2022) drew attention to the importance of learning-by-doing in the pricing context, drawing from the evidence of recently deregulated markets to show how pricing strategies converge over time to something like the optimal plan as firms learn what works best. Similarly, Felzensztein et al. (2022) found that more experienced management teams help international market entry, which pays off mainly for larger SMEs due to the interaction with the company’s resources.

Of the various business processes needed for success, many authors consider learning about technology and its optimal use especially important. An OECD report (1996) looking at the knowledge-based economy argued that essential areas of knowledge such as ‘know-how’ and ‘know-who’ are rooted primarily in practical experience and that appropriate use of IT can aid this. It is also argued that ‘learning-by-doing’ is paramount in a knowledge-based economy. Though that report precedes the advent of social media sites, the general point seems even more accurate today. Other literature to support this idea includes Shih and Venkatesh (2004), who considered how new technologies (social media in this case) are spread throughout society. They developed a theory of ‘use-diffusion’ for new technology. This theory posits that both the rate and variety of use of new technology are essential dimensions and that users can be placed in different categories based on how they use new technology. Experience using the technology is put forward as a critical characteristic in how new technology is used. Their empirical study found that higher accumulated experience using the latest technology significantly affects its variety and rate of use. A similar finding comes from research by Jones et al. (2015), who find that social media can benefit a firm but that there are barriers to using it effectively. One of these barriers is a lack of experience and knowledge in using social media, and the authors advocate a role for training to aid business owners. Investing time in learning the effective use of a specific social media platform is crucial for small businesses.

In summary, an essential method for gaining knowledge about the proper use of social media is likely via ‘learning-by-doing’. Therefore, we propose a second hypothesis:

Hypothesis 2 (H2): Knowledge of social media is facilitated by learning through experience or explicit training.

For businesses that find social media helpful in increasing sales, there will still be a variance between those for whom social media is essential and those for whom it is less so.

In some industries and for some firms, social media presence may be more essential; for example, new entrants into a market may require more promotion to gain consumer awareness. Generating a social media presence is a necessary part of this.

Where a firm considers that social media is essential, there will be more incentive to invest more time and effort into producing their social media presence, which will enhance the positive effect on sales that social media can have. Bogea and Brito (2018) found that several variables affect social media adoption based on interviews with company executives. These include knowledge of social media (supporting Hypothesis 1) and the customer’s presence on social media. This suggests that for companies with a customer base that is more highly engaged with social media, the development of a social media presence is likely to be more critical. Similar reasoning can be found in the paper by Durkin et al. (2013), who conducted an in-depth study into the social media strategy of several companies. These companies were self-selected for the study based on their perceived need to develop their social media presence. In most cases, this was further reinforced by customers’ perceived high level of interest in creating a more significant social media presence.

Brandao et al. (2019) found that social media can be crucial in facilitating access to international markets by allowing the establishment of an emotional connection. This link to internationalisation is further supported by Amorós et al. (2014), who generally linked the adoption of new technologies to better firms’ access to international export markets. Their specific focus is on neither brand-new nor very established technology. They interpreted that using new technology requires skill and effort to create the advantage that allows the firm to compete successfully in an international environment (again supporting Hypothesis 1). This point on the success of internationalisation is supported by Aronica et al. (2021), who found that small firms that remain at a basic level of social media use are also less likely to enter international markets. There are, therefore, several reasons to think that some firms will perceive that a well-developed social media presence is of particular importance, perhaps due to their sector, the nature of their consumer base, or the desire for internationalisation, among other possible factors. Therefore, we develop the third hypothesis:

Hypothesis 3 (H3): The more important social media adoption is to a firm’s business strategy, the more SMEs will adopt social media.

## Survey design

The survey to explore how SMEs in Kenya and Nigeria use social media, and its impact on sales, was conducted in 2016 as part of a faculty seed fund project by De Montfort University. The faculty seed fund project aimed to discover new cross-disciplinary and international research areas.

The survey we administered applies economic theory to technology change by interrogating SMEs’ use of social media as a business strategy in developing countries. Consequently, the survey result identifiers that influence social media adoption across two countries tested H1, H2, and H3.

The qualitative questions were split into three parts. The first part of the questionnaire gives us insight into how much time firms spend using social media for business purposes, which is linked to H2. The second part of the questionnaire provides insight into their motivations for using social media as a business strategy. This helps to build a picture of the importance of using social media for SMEs and to test H3.

Quantitative questions were used to gain statistical results on the likelihood that the time and knowledge invested in building a social media presence increased sales. The responses from the quantitative questions were used to develop constructs for testing understanding of social media to test H1. The survey helped us to capture two dimensions of social media: (i) understand why and how local businesses use social media and (ii) understand from the perception of local businesses in Kenya and Nigeria the leverage social media has on sales.

### Data collection and sample

After briefly explaining the study objectives, 100 local SMEs in Lagos (Nigeria) and Nairobi (Kenya) were administered a questionnaire.

The firms selected for the survey were those with experience using social media. Therefore, we assumed that firms in Lagos and Nairobi are more likely to use social media than firms in relatively less commercial regions. This is due to access to facilitation amenities such as reliable internet connectivity.

In Nairobi, the survey was carried out by three local research assistants hired to distribute the survey and collect responses from these firms. The research assistants were trained in the survey questionnaire. In Lagos, the questionnaires were distributed by two professional surveyors with the assistance of the Advertising Practitioners Council of Nigeria (APCON)^1^. APCON’s assistance provided us with links to numerous business enterprises through their agents, who were instrumental in the questionnaires’ delivery, completion, and collection. We ensured that both the research assistants and professional surveyors in Lagos understood each question in the survey. This activity helped to reduce misunderstandings and, therefore, incorrect responses from firms. The questions used in the survey can be found in the online appendix. The survey results are outlined below and correspond to the pertinent sections of the questionnaire.

#### Characteristics of the surveyed SMEs (Q1–3)

According to the OECD, an SME is defined as having fewer than 250 employees. In Kenya and Nigeria, respectively, most enterprises surveyed were mainly SMEs operating for less than 10 years.

The fact that most SMEs surveyed in Kenya and Nigeria are less than 10 years old may explain why they use social media. SMEs, especially those less established in terms of years of operation, use social media to compete with larger enterprises in reaching customers and gaining potential new customers. This is in light of observations that social media has taken flight in the last decade (Carter, 2014), mainly to enable enterprises to keep up with their competitors, as using social media has become fashionable (Cesaroni and Consoli, 2015).

#### Use of social media (platform) (Q7)

**Fig. 1:**
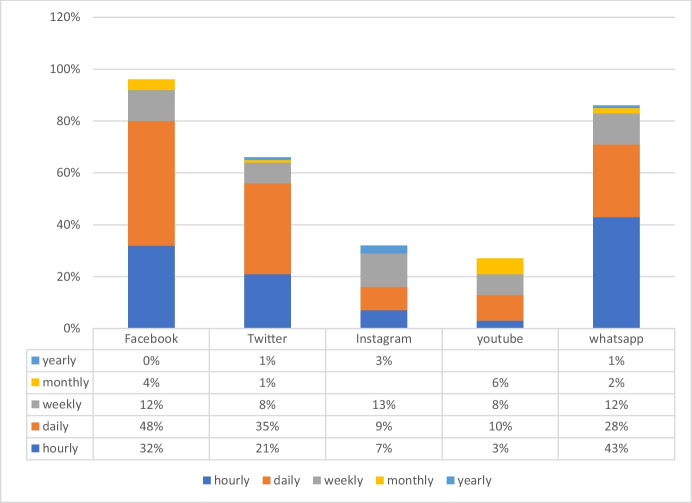
Frequency of use of social media in Nairobi

**Fig. 2:**
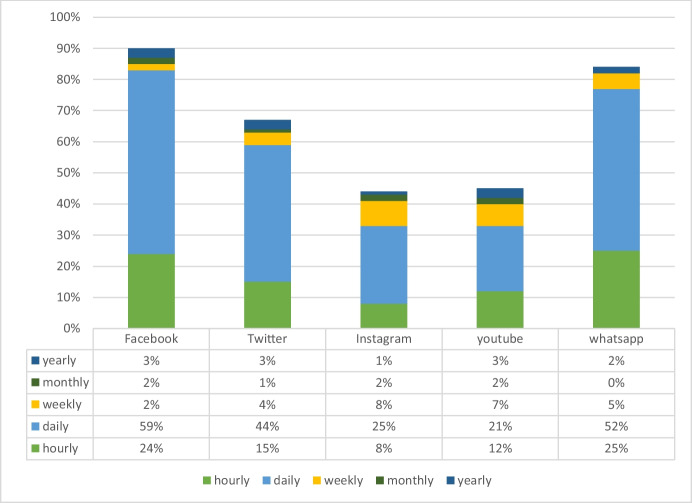
Frequency of use of social media in Lagos

Figures 1 and 2 present enterprises’ responses to social media use in Kenya and Nigeria, respectively. The results show that Facebook, Twitter, and WhatsApp were the most popular social media platforms used by enterprises in Kenya. When asked how regularly they used social media, WhatsApp and Facebook were the most popular social media platforms. On an hourly basis, 43% of firms responded that they visited WhatsApp, while 32% answered that they visited Facebook. The evidence suggests that most SMEs sampled in the survey spend significant time on social media.

Figure 2 shows that 25% of SMEs in Lagos use WhatsApp hourly, followed by Facebook (24%). The evidence lends credence to the hypotheses (H1 and H2) that spending time on social media is crucial if firms want to use social media to increase sales.

#### Why SMEs use social media (Q. 11)

The survey asked why enterprises in both Lagos and Nairobi used social media. About 90% of the enterprises in Kenya and Nigeria mainly use social media for advertising products or services. Furthermore, about 80% of the SMEs surveyed in both countries cited gathering information from customers about their products as the second reason for using social media further. This reveals that SMEs use social media to help them develop market knowledge. The survey responses concur with a recent study by Aronica et al. (2021) which found that firms are more likely to use social media for strategic targets, such as marketing or getting customer feedback. Consequently, knowing how to use social media for advertising is a crucial key business strategy for firms to leverage sales.

## Data

For empirical application, the measure we chose to focus on was using social media to increase sales. The reason for this is that, when conducting the survey, we found that the essential function for firms is advertising, which is to increase sales. Second, as previous literature indicates (Adewale et al. 2013), firms should be able to measure better, or at least have a feel for, how sales may change in response to changes in business strategy. Across the sample, the percentage of firms who either strongly agreed or agreed that social media is used to promote sales in their business was 75% in Kenya and 94.9% in Nigeria.

Several questions in the survey contribute to measuring the role of learning-by-doing, and so can be used to test H2. One question in the survey captures the amount of time invested in using social media. It asks firms how regularly they use social media platforms for business purposes. The survey results showed that SMEs use all social media platforms (i.e. Facebook, WhatsApp, Twitter, Instagram, YouTube) in Lagos and Nairobi. Facebook was the most common platform used by SMEs to advertise their businesses. Figures 1 and 2 show that 80% of SMEs in Kenya and 84% of SMEs in Nigeria use Facebook hourly and daily.

The key indicator for use in testing H1 is knowledge. The survey interrogated firms on their understanding of using social media to gather information about their posts. This information included (i) number of hits, (ii) number of likes, (iii) product ratings, (iv) sales level, and (v) revenues per customer. We then constructed the variable ‘knowledge’ from the data set 𝐾𝑖, which ranks the firms’ knowledge of using social media platforms in ascending order, i.e. poor knowledge = 1, moderate knowledge = 2, and good knowledge = 3. Firms that were exceptionally confident of finding at least three of the above information were ranked as having ‘good knowledge of using social media tools’ and received the highest ranking for 𝐾𝑖. Firms that expressed some knowledge of using social media to find information were ranked as having a moderate understanding of social media. Finally, firms that did not know how to use social media tools to find information were classified as having poor knowledge and given the lowest ranking.

To test the reliability of the responses for 𝐾𝑖, Cronbach’s *α* was estimated; the reliability coefficients for 𝐾𝑖 are 0.858 and 0.803 for Kenya and Nigeria, respectively, and are above the minimum threshold of 0.6 for social sciences research (Anderson and Gerbing, 1988).

### Empirical strategy

We combined the two primary data sources from Kenya and Nigeria to create a sample for quantitative analysis. The empirical analysis follows the approach of Islam et al. (2018), where samples for Kenya, Tanzania, and Uganda were pooled to analyse the relationship between investment and mobile money. To model the research question and test the hypotheses, we estimated the following equation:

1 $${S}_{ij}={\alpha}_0+{\alpha}_1{K}_{ij}+{\alpha}_2{B}_{ij}+{\alpha}_3{H}_{ij}+{\alpha}_3 Ag{e}_{ij}+{\alpha}_4 Siz{e}_{ij}+ Countr{y}_j+{\varepsilon}_{ij}$$

where 𝑆𝑖𝑗 is a binary variable that takes a value of 1 if the responder agrees that social media is used to promote sales for firm *i* in country *j*, and 0 otherwise. Therefore, 𝑆𝑖𝑗 proxies for social media adoption. 𝐾𝑖 is knowledge of social media and relates to H1. It represents how confident firms were in using any social media platform to find information, as described in the ‘Data’ section. 𝐵𝑖 is the importance of social media as a business strategy and relates to H3. 𝐻𝑖 is time invested in learning how to use social media and refers to H2. Facebook is used as a proxy for social media platforms. The control variables are the age of the business (𝐴𝑔𝑒) and firm size (𝑆𝑖𝑧𝑒). 𝐴𝑔𝑒 is a dummy variable that takes the value of 1 if the firm has existed for more than 10 years and 0 otherwise. 𝑆𝑖𝑧𝑒 is a dummy variable that takes a value of 1 if the firm employs more than 250 people and 0 otherwise. Felzensztein et al. (2022) is among the authors finding that firm size plays a role in moderating the role of experience and knowledge in business success, so this variable is essential to control for. Finally, we include a dummy variable for 𝐶𝑜𝑢𝑛𝑡𝑟𝑦 where 0 = Kenya and 1 = Nigeria. 𝜀𝑖𝑗 is an error term. Table 1 reports the list of variables selected for analysis and presents the descriptive statistics. These variables were either taken directly or constructed using the responses from the survey.

In the model for estimation, there is a potential bias in the results because the dependent variable (whether social media is used to promote sales) and time invested in social media could be interpreted to measure social media use. We then estimated the relationship between social media adoption and learning-by-doing in two stages. In stage 1, we modelled how learning-by-doing occurs by showing that the accumulation of knowledge of social media comes in part from the time invested in learning how to use social media. In stage 2, we modelled that social media adoption is influenced by knowledge of how to use social media. The estimation method is given in Eqs. 2 and 3, where 𝑍𝑖𝑗 are the control variables for firm *i* in country *j*.

2 $$\textrm{Stage}\ 1:{K}_{ij}={\beta}_0+{\beta}_1{H}_{ij}+{\alpha}_2{B}_{ij}++{\beta}_3{Z}_{ij}+ Countr{y}_j+{\varepsilon}_{ij}$$

3 $$\textrm{Stage}\ 2:{S}_{ij}={\gamma}_0+{\gamma}_1{K}_{ij}+{\gamma}_2{B}_{ij}+{\gamma}_3{Z}_{ij}+ Countr{y}_j+{\varepsilon}_{ij}$$

The advantage of this estimation strategy is that it pins down social media adoption as firms’ use of social media to promote sales.

We estimated Eq. (2) as the first stage of our analysis. The results confirm that time invested in learning how to use social media significantly increases the accumulation of knowledge in social media.^2^ The results are consistent with the learning by doing evidence of Irwin (2000) and H2.

## Results and discussion

Table 2 presents the second stage of the analysis that social media adoption is significantly influenced by knowledge of how to use social media and supports H1 and H2. This is consistent with the evidence by Jones et al. (2015) that knowledge is an essential factor in adopting social media among firms. The results in Table 2 also analysed the relationship between social media adoption, learning-by-doing, and the strategic business importance of involvement with social media. We used a binary logistic regression model for estimation because social media adoption (dependent variable) takes a value of 1 if firms use social media to promote sales and 0 otherwise. Consequently, the coefficients in Table 3 are odds ratios that interpret the likelihood of occurrence. The standard errors are in parentheses.

**Table 2:** Factors that influence social media adoption

*Dependent variable: Is social media used to promote sales in your business?* *where 0 = No and 1 = Yes*		
Variables	Exp (*β*)	Exp (*β*) Clustering around firm size
	Logit Odds ratio (se)	Logit Odds ratio (se)
Knowledge of social media	2.89*** (0.997)	2.89*** (0.126)
Business strategy importance	3.78*** (1.61)	3.78*** (0.324)
Age	0.95 (0.42)	0.946 (0.420)
Size	1.31 (0.83)	1.32*** (0.075)
Country	0.224*** (0.099)	0.224*** (0.002)
Wald chi^2^ (5)	26.08	-
Prob>chi^2^	0.001	-
Wald chi^2^(5)	26.08	-
Prob>chi^2^	0.001	-
No. of observations = 159		
*R*^2^ = 0.23		

* is significant at 10% level; ** is significant at 5% level; *** is significant at 1% level

**Table 3:** Robustness tests: factors influencing social media adoption by firm size

*Dependent variable: Is social media used to promote sales in your business?* *where 0 = No and 1 = Yes*				
Variables	Logit		OLS	
	*SME*^′^*s*	Large	*SME*^′^*s*	Large
Knowledge of social media	2.75*** (0.062)	1.70 (1.41)	0.18*** (0.062)	0.25 (0.28)
Business strategy importance	3.91*** (1.74)	1.39 (1.31)	0.25*** (0.078)	0.12 (0.225)
Age of business	0.896 (0.415)	−1.31 (1.09)	0.002 (0.074)	−0.13 (0.15)
Country	0.209* (0.095)	−1.67 (1.66)	−0.24*** (0.068)	−0.20 (0.17)
Wald chi^2^(4)	25.45	3.12	F(4,139) = 14.19	F(4,18) = 0.82
Prob>chi^2^	0.000	0.538	Prob>4 = 0.00	Prob>F = 0.5272
*R*^2^	0.23	0.14	0.27	0.12
No. of observations	144	23	144	23

* is significant at 10% level; ** is significant at 5% level; ***is significant at 1%

Firms that ranked social media as key to their business strategy were four times more likely to adopt social media. This supports the third hypothesis, H3. According to Gligorijevic and Leong (2011), some of the SME organisational objectives of using social media include micro-blogging, such as on Facebook for brand awareness, online advertising, and developing an online communications network. These strategies can help SMEs generate leads and increase sales. Most of the firms surveyed in Kenya were from the service sector, where social media is a vital marketing tool to reach customers (Öztamur and Karakadılar, 2014). On the other hand, most of the firms surveyed in Nigeria were classified as ‘miscellaneous’ sectors. These are firms that produce a wide range of products and personal services. Consequently, firms in these sectors highly rank social media as a key business strategy, both now and in the future. The survey showed that 95% of firms in Kenya and 86% of firms in Nigeria would continue to use social media to promote their businesses.

We now consider the results of the control variables that capture firm characteristics. Age is a positive but not significant factor in the adoption of social media. In the second column of Table 3, we controlled for clustering effects according to firm size because 80% of the firms in the sample are SMEs. OLS regressions assume that residuals are independent. Although we assumed that firms across the two countries are independent, there may be some correlation in the responses on the use of social media, which could lead to residuals between small and large firms not being independent. Regardless of the firm’s size, firm owners or a hired specialist use common technology to access social media platforms such as mobile phones or PCs. The results showed that the estimates between the two columns are broadly similar, but the standard errors consider observations on social media use between large and small firms that are non-independent. As a result, size was positive and significant, showing that it is a factor that influences the likelihood of adopting social media. Although social media is more prevalent among smaller firms, larger firms can invest more in technology because they have more resources and networks (Qalati et al., 2022; Cull and Xu, 2005). Finally, the country dummy showed that firms in Nigeria are more likely to adopt social media than in Kenya. This is not surprising, as the survey showed that firms in Nigeria were more strongly in favour of using social media than in Kenya.

### Robustness checks: SMEs vs large firms

Braojos-Gomez et al. (2015) showed that using social media is more important for smaller firms than for larger firms because they have limited financial resources. The literature indicates that among SMEs, social media is a vital instrument in connecting enterprises and customers, enterprises and enterprises, and between customers. Chatterjee and Kuman Kar (2020) explained that social media has been influential in the business affairs of SMEs, such as in business growth.

Table 3 presents the results of the factors influencing the social media adoption split between SMEs and large firms. We found that SMEs are approximately three times more likely to adopt social media when their knowledge of social media tools is better. Furthermore, SMEs were four times more likely than larger firms to embrace social media as a business strategy within the organisation. Both variables are significant at the 1% level.

The results were consistent with evidence by Meske and Stiglitz (2013) that smaller firms are more likely to be involved in social media than larger firms, which tend to use more traditional marketing channels. In comparison, the odds ratios of knowledge of social media and business strategy importance were positive but insignificant for large firms. Given the small size of the large firms in the survey, the overall model’s fit was not statistically significant. We proceeded to re-estimate the models using ordinary least squares and found similar results.

### Implications of the results

These results generally reflect how internet-based technologies have changed how SMEs conduct business. Social media adoption among SMEs is an exciting concept because entrepreneurs in developing countries can use e-commerce in various business functions, including the possibility of accessing international markets.

From this analysis, we can draw the following implications: The results have shown a large capacity among service-based urban SMEs in Kenya and Nigeria to adopt social media. This is consistent with evidence from Fernandes et al. (2016), who found that service-based SMEs in Portugal were more likely to embrace social media. However, these results cannot be generalised to a different industry context or rural areas facing more technological barriers.

The results show that social media adoption increases with more time invested and knowledge in using the technology. The implication is that access to international markets for SMEs in developing countries is difficult without technological infrastructure and increased digital literacies. Furthermore, SMEs with a basic knowledge of social media face an obstacle in economic benefitting from the technology. Policymakers need to increase digitisation through the educational system to enable young and aspiring entrepreneurs to internationalise as part of their entrepreneurial and strategic orientations. Ensuring good-quality digital infrastructure is also of increasing importance. At the firm level, entrepreneurs should ensure they familiarise themselves with the fundamental features of social media sites and work to transfer their knowledge from personal use into their business life.

### Academics and practitioners

From an academic perspective, our article highlights the role of learning and knowledge of social media as a cause of internationalisation among firms, particularly SMEs, to compete with their larger counterparts. Understanding the use of social media as a strategy to drive internationalisation is currently at the early stages of research. Further research on the role of knowledge in amplifying the use of social media as a business tool will help determine the channels by which social media contributes to increasing sales.

For practitioners, our article provides a starting point for understanding aspects of how SME entrepreneurs in developing countries use social media to internationalise their business operations. Further research could focus more intensely on different types of social media SMEs use. This technology is used daily to rapidly broaden their business by increasing their visibility in international markets.

### Limitations

The implication of our results, which emphasise the role of time and knowledge as factors that influence social media adoption, are exclusive to our data range and do not cover other factors that may be present in other regions, such as access to reliable networks, electricity, computers, devices, or experts. Due to limited resources, we did not consider social adoption in cities outside Lagos (Nigeria) and Nairobi (Kenya). Therefore, these results cannot be generalised to rural areas with more technological barriers. Consequently, a more extensive study to analyse social media adoption among SMEs in developing countries would enable comparing SMEs in urban vs rural areas. Second, the survey covers a short period using 100 usable responses in Nairobi and 99 usable responses in Lagos. This means the results reveal a snapshot of the factors that influence social media adoption at a point in time. As such, there is a need in the future to conduct a more extensive study over a longer duration. Finally, our analysis does not identify the factors that influence the non-adoption of social media.

## Conclusion

This study has conducted a primary survey of local SMEs in Kenya and Nigeria to gauge business attitudes toward using social media in their business. The survey explored the extent to which SMEs use social media in their business, providing a crucial contribution to the wide gap between theory and practice in social media adoption by showing the role played by learning-by-doing among SMEs in developing countries. It reveals the aspirations of SMEs in developing countries to know how social media can bring more international exposure to their business and increase customer reach.

The results have shown that social media adoption by firms increases as more knowledge is invested in developing a social media presence. Furthermore, firms that rank social media highly as a business strategy are likelier to use it for business promotion. Finally, the results support the broader literature that the use of social media is vital to smaller firms in strategically connecting with cross-border markets.

## Supplementary information

ESM 1. (DOCX 13 kb)

## Data Availability

Dataset used in analysis using STATA is available.

## Footnotes

1 APCON is a government agency whose role is to vet advertisements by organisations, companies, or individuals before they are released to the public.

2 Results available and can be requested from the author
