# Supplementary material for: Analysing the factors that influence social media adoption among SMEs in developing countries
Source: J Int Entrep. 2023 Apr 25:1–20. Online ahead of print. doi: 10.1007/s10843-023-00330-9 (PMC10127956; doi:10.1007/s10843-023-00330-9)
Supplement: Supplementary file 1 — (DOCX 13 kb) [file 10843_2023_330_MOESM1_ESM.docx]

**Appendix A: Summary Survey questions**

- Q1: What industry is your business involved in?
- Q2: When was the business started?
- Q3: How many people are employed in this business?
- Q.6: Do you use the following social media tools for business purposes? (Please tick all that apply: Facebook, Instagram, Twitter, YouTube, LinkedIn, Google Analytics.)
- Q.7: How regularly do you use the following social media tools to promote your business? (Please tick all that apply: Hourly, Daily, Weekly, Monthly, Yearly.)
- Q.8: Who uses social media in your business? (myself, another member of staff)
- Q.10: How important is the use of social media to your business strategy? (Very important, important, somewhat important, very unimportant)
- Q.11: Why do you use social media in your business? (Please tick all that apply: Advertise product/service, gather information from customers, link with other businesses, product reviews, others.)
- Q.12: Has social media increased sales in your business? (Strongly agree, agree, neither agree nor disagree, disagree, strongly disagree)
- Q.14: Has social media allowed you to better understand your customers? (Strongly agree, agree, neither agree nor disagree, disagree, strongly disagree)
- Q.24: How confident are you in using social media to find out the following information: (i) Number of hits/visits/page reviews, (ii) Number of followers/friends, (iii) Number of likes/dislikes, (iv) product ratings, (v) sales level, (vi) revenue per customer? (Very confident, a little confident, not confident at all)
- Q.25: How often do you use social media to find out the: (i) number of hits/visits/page reviews, (ii) number of followers/friends, (iii) number of likes/dislikes, (iv) product ratings, (v) sales level, (vi) revenue per customer? (Frequently, sometimes, seldom, never)

N.B Full Survey are available from authors upon request
